# Elevated Serum Homocysteine Levels Have Differential Gender-Specific Associations with Motor and Cognitive States in Parkinson's Disease

**DOI:** 10.1155/2019/3124295

**Published:** 2019-05-29

**Authors:** Megan C. Bakeberg, Alexa Jefferson, Maddeson Riley, Michelle Byrnes, Soumya Ghosh, Frank L. Mastaglia, Malcom K. Horne, Sarah McGregor, Rick Stell, Jade Kenna, Sue Walters, Dana Hince, Ryan S. Anderton

**Affiliations:** ^1^Perron Institute for Neurological and Translational Science, Nedlands, WA, Australia; ^2^Centre for Neuromuscular and Neurological Disorders, University of Western Australia, Nedlands, WA, Australia; ^3^School of Health Sciences, University of Notre Dame Australia, Fremantle, WA, Australia; ^4^Florey Institute for Neuroscience and Mental Health, University of Melbourne, Parkville, Victoria 3010, Australia; ^5^Centre for Clinical Neurosciences and Neurological Research, St Vincent's Hospital Melbourne, Fitzroy, Victoria, 3065, Australia; ^6^Institute for Health Research, University of Notre Dame Australia, Fremantle, WA, Australia

## Abstract

**Background:**

Studies attempting to elucidate an association between homocysteine and symptom progression in Parkinson's disease (PD) have had largely discrepant findings. This study aimed to investigate elevated serum homocysteine levels and symptom progression in a cohort of PD patients.

**Methods:**

Serum homocysteine, folate, and vitamin B12 levels were measured in 205 people with PD and 78 age-matched healthy controls. People with Parkinson's disease underwent a battery of clinical assessments to evaluate symptom severity, including motor (MDS-UPDRS) and cognitive (ACE-R) assessments. Multivariate generalised linear models were created, controlling for confounding variables, and were used to determine whether serum markers are associated with various symptom outcome measures.

**Results:**

People with Parkinson's disease displayed significantly elevated homocysteine levels (*p* < 0.001), but not folate or vitamin B12 levels, when compared to healthy controls. A significant positive correlation between homocysteine and MDS-UPDRS III score was identified in males with Parkinson's disease (*r*_*s*_ = 0.319, *p* < 0.001), but not in females, whereas a significant negative correlation between homocysteine levels and total ACE-R score was observed in females with Parkinson's disease (*r*_*s*_ = −0.449, *p* < 0.001), but not in males. Multivariate general linear models confirmed that homocysteine was significantly predictive of MDS-UPDRS III score in male patients (*p*=0.004) and predictive of total ACE-R score in female patients (*p*=0.021).

**Conclusion:**

Elevated serum homocysteine levels are associated with a greater motor impairment in males with Parkinson's disease and poorer cognitive performance in females with Parkinson's disease. Our gender-specific findings may help to explain previous discrepancies in the literature surrounding the utility of homocysteine as a biomarker in PD.

## 1. Introduction

Parkinson's disease (PD) is a chronic and progressive neurological disease that is characterised by the onset of an array of motor and nonmotor signs and symptoms. Nonmotor symptoms, including cognitive impairment, apathy, emotional disturbance, and sleep disturbance, are commonly reported as being equally as debilitating as cardinal motor symptoms [1]. It is well established that the progression and clinical symptom presentation varies considerably among people with Parkinson's disease (PwP) [2], with some studies indicating that several nonmotor features of PD often precede traditional motor signs [3]. Despite the well-characterised symptomatology of this disease, the specific pathogenic mechanisms underlying the death of such a vast array of neurons and structures cannot yet be explained. Therefore, factors that predict these varying outcomes in PwPs and potential diagnostic biomarkers of this degenerative disease require exploration.

Homocysteine (Hcy) is a thiol-containing, nonessential amino acid that is generated in all cells as a by-product of methionine and folate metabolism [4, 5]. Hcy is normally metabolised through two biochemical pathways, during remethylation to methionine and transsulfuration to cystathionine [6]. Therefore, Hcy can accumulate if these biochemical processes become dysregulated [6, 7]. Typically, elevated Hcy levels are associated with increasing age, male gender, caffeine consumption, lack of physical activity, and smoking [7, 8]. Additionally, low levels of dietary vitamin B12 and folate have been associated with elevated serum Hcy, most likely due to the role they have in Hcy metabolism [9, 10]. High levels of serum Hcy, known as hyperhomocysteinemia (HHcy), are thought to contribute to endothelial dysfunction and oxidative damage [7, 11–15]. While previous studies have implicated high levels of serum Hcy in stroke and other cardiovascular disorders [16–18], associations with neurodegenerative disorders such as dementia and Alzheimer's disease (AD) have also been identified [9, 19].

Several studies have reported an association between HHcy and PD; however, such associations have been thought to be a result of long-term levodopa (L-DOPA) therapy [20, 21]. Interestingly, these studies have also indicated that L-DOPA, itself, may be the principal cause of elevated Hcy levels, as opposed to a consequence of the disease [5, 20–22]. Many studies indicate that elevated Hcy levels can occur independently of PD medication due to genetic variants and nutritional deficiencies of vitamin B12 and folate [23, 24]. Further to this, it is thought that these variants or deficiencies can lead to greater susceptibility to HHcy following levodopa treatment, which is a risk factor for more rapid cognitive decline and progression of motor impairment [20, 25, 26]. To date, investigations into the relationship between elevated Hcy and rate of PD progression and severity have yielded inconsistent results, with variation occurring in studies reporting whether or not HHcy is a risk factor for disease progression [2, 20, 25]. As such, it remains unclear whether HHcy is a significant contributor to PD, or whether disease progression leads to the elevation of Hcy levels [12]. Similarly, the association between elevated levels of Hcy and cognitive dysfunction remains unclear. Findings from a number of studies suggest that elevated Hcy may contribute to the development and exacerbation of cognitive impairment in PD [5, 15, 24, 27–29], and others have reported no association between Hcy serum levels and cognitive impairment [24, 30].

Despite extensive research, there are still inconsistent results surrounding the association between HHcy and clinical features of PD, and therefore the utility of Hcy as a biomarker in PD remains in question. As such, this study aimed to investigate homocysteine levels in a cohort of PwP and healthy controls and determine the effect of elevated serum Hcy on patient motor and cognitive performances.

## 2. Methods

### 2.1. Participants

Two-hundred and five home-based PwP (128 males and 77 females) and 78 (31 males and 47 females) aged-matched healthy controls were sequentially recruited from Movement Disorders Clinics at the Perron Institute for Neurological and Translational Science (Perth, Australia), St. Vincent's Hospital (Melbourne, Victoria), and Royal North Shore Hospital (Sydney, New South Wales), between 2012 and 2015. All PwP were examined by a movement disorder neurologist prior to inclusion in the study for verification of the diagnosis in accordance with the UK Brain Bank criteria for idiopathic PD [31]. This study was approved by a Human Research and Ethics Committee (Approval number 2006/073), and written informed consent was obtained from all participants, in accordance with the National Health and Medical Research Council guidelines.

### 2.2. Clinical Assessments of PwP

Clinical evaluations included collection of patient demographic variables and medication dosage, assessments of motor and cognitive function, and other disease-related features (Table 1). All PD medications were converted to a total levodopa equivalent dose (LED), based on a previously reported conversion equation [32, 33]. Motor symptoms were evaluated in the “ON” state using the Movement Disorder Society-Unified Parkinson's Disease Rating Scale (MDS-UPDRS) Part III and Hoehn and Yahr (H&Y) Scale [34]. In addition, each participant was evaluated by a clinical psychologist and completed a panel of neuropsychological assessments, as previously described [35]. Global and domain cognitive function was assessed using the revised “Addenbrooke”s Cognitive Examination' (ACE-R) [36, 37].

### 2.3. Serum Analysis

Fasted patient and healthy volunteer control blood samples were collected prior to clinical or psychological assessments. For blood collection, 10 ml of whole blood was taken from a median cubital vein and stored in a standard BD EDTA vacutainer® (Becton Dickinson and Company, Franklin Lakes, N.J.). Serum Hcy, vitamin B12, and folate were recorded for analysis in this study.

### 2.4. Statistical Methods

Data were analysed using IBM-SPSS (v. 24, IBM Corporation). Where appropriate, univariate regression analysis or Mann–Whitney *U*-test was performed to identify differences between patient and control serum markers. Cohen's d ESs were calculated for the mean differences, with an ES of 0.20 considered small, 0.50 medium, and 0.80 large. Spearman Rho correlation coefficients were used to assess the relationship between serum Hcy level and continuous patient-related variables, with values of *r* = 0.10 considered small, 0.30 medium, and 0.50 large. Generalised linear models (GLM) were used to investigate outcome measures, such as motor severity and cognitive score, with serum Hcy as an independent variable. GLMs were used to analyse the relationship between variables identified as being significant in the univariate models, the motor symptoms assessed in the MDS-UPDRS part III examination, and the cognitive score assessed in the ACE-R. Variables proposed to be risk factors for the development of high serum Hcy levels in PD were also included in the GLMs. Variables included in the GLMs were as follows: age at assessment, LED, DBS history, and disease duration. A significant nominal *p* value of ≤ 0.05 was employed.

## 3. Results

### 3.1. Homocysteine Levels Are Elevated in Males and Patients with PD

**Figure 1:**
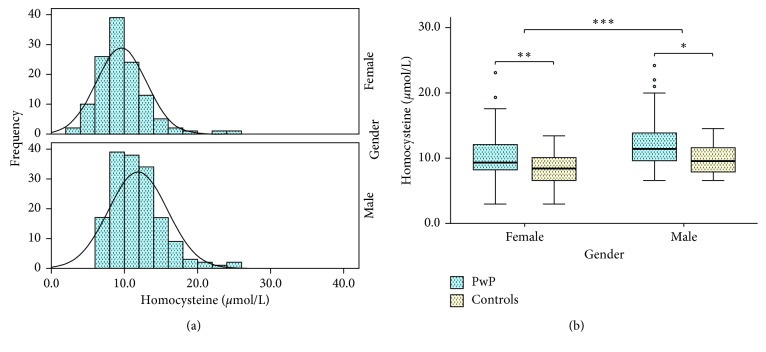
Comparison of serum Hcy levels in PwP and healthy, age-matched controls. Average levels of Hcy were significantly higher in males than females (a). Levels of serum Hcy were significantly higher in PwP compared to healthy controls, in both males and females (b). Data are presented as mean ± SEM. ^*∗*^ *p* < 0.05; ^*∗∗*^ *p* < 0.005; ^*∗∗∗*^ *p* < 0.001.

Overall, the distribution of Hcy levels differed in males and females, with the average serum level in males being higher in both patient and control samples (Figure 1(a)). Mean comparisons revealed significant differences in serum Hcy in males and females with PD (Figure 1(b); *p* < 0.001 and *p*=0.009, respectively; *d* = 0.629). Furthermore, mean comparisons revealed significant differences in serum vitamin B12 in males with PD (Table 2, *p* < 0.001), but the same could not be said for females with PD (Table 2, *p*=0.163). Such differences occurred independently of any significant changes between serum folate (*p*=0.133) or vitamin B12 levels (*p*=0.054).

### 3.2. Gender-Specific Correlations with Hcy Levels in PwP

Given the observed gender differences, male and female PwPs were analysed separately. Spearman's rho analysis revealed a significant positive correlation between patient age and Hcy level in both males (*r*_s_ = 0.163, *p*=0.037) and females (*r*_s_ = 0.366, *p* < 0.001). In addition, LED (*r*_s_ = 0.278, *p*=0.001) and disease duration (*r*_s_ = 0.230, *p*=0.008) were significantly correlated with Hcy levels in male patients, but not in female patients.

In male patients, a significant positive correlation between Hcy levels and MDS-UPDRS III (*r*_s_ = 0.319, *p* < 0.001) was observed. In contrast, there was no significant association between Hcy levels and MDS-UPDRS III scores in females (Table 3). Conversely, in female patients, Hcy levels were significantly inversely correlated with cognitive scores, as indicated by the ACE-R total score (*r*_s_ = −0.449, *p* < 0.001), whereas there was no association between Hcy levels and cognitive scores in male PD patients (Table 3).

### 3.3. Elevated Homocysteine Levels Significantly Associate with MDS-UPDRS III Scores in Males

To determine if Hcy levels were predictive of motor symptom severity, multivariate general linear models (GLMs) were used. Identified correlates of Hcy levels, such as age at assessment, disease duration, and LED, were included in final models. When controlling for these variables, serum Hcy levels were predictive of elevated MDS-UPDRS III scores in males (*p*=0.004; Table 4). Specifically, for each additional micromole per litre unit (*µ*mol/L) of serum Hcy, male patients were predicted to score 0.77 points higher on the MDS-UPDRS III. However, serum Hcy levels did not significantly associate with MDS-UPDRS III scores in females, with females only predicting to score 0.375 points higher on the MDS-UPDRS III for every *µ*mol/L of serum Hcy. Final GLM models indicate that when controlling for potential covariates, Hcy levels are significant predictors of motor outcome/severity scores in males, but not females.

### 3.4. Elevated Homocysteine Levels Significantly Associate with ACE-R Cognitive Score in Females

When controlling for disease duration, age at assessment and LED, serum Hcy was predictive of total ACE-R score (*p*=0.021; Table 5). Specifically, for each additional *µ*mol/L of serum Hcy, female patients were predicted to score 0.47 points lower on the ACE-R assessment. However, the same could not be said for male participants, who only saw a 0.016-point decrease for total ACE-R scores, which is approximately 90% less of a change than seen in females. Final GLM models indicate that when controlling for potential covariates, elevated Hcy levels are a significant correlate of decreased cognitive function in females, but not males.

## 4. Discussion

Elevated serum Hcy is reported as a risk factor for stroke and other cardiovascular disorders [16–18], with mixed findings in PD [38]. In the present study, we report that gender plays a significant role in determining the effects of Hcy as a predictor for motor and cognitive outcome measurements in PD. Specifically, it appears that female patients have an inverse association between serum Hcy and cognitive functioning, whereas male patients have an inverse association between serum Hcy and motor ability.

Although the evidence is somewhat inconsistent, gender differences in serum Hcy have been documented in PD, as well as other disorders [39]. For instance, Wang et al. [40] found that females with cardiovascular diseases or acute pancreatitis had normal Hcy levels (5–15 *μ*mol/L), whereas their male counterparts averaged Hcy levels 5 *μ*mol/L higher [40]. The current study produced similar findings, whereby male participants, regardless of disease severity, had significantly higher Hcy levels than their female counterparts (*p* < 0.001; *d* *=* 0.450). The observed gender differences in serum Hcy may be explained by a variety of factors, including the effects of sex hormones during and postmenopause in women, larger muscle mass in men, and lifestyle factors such as diet and smoking [40–43]. In recent studies, serum Hcy levels in males and females have been compared throughout different stages of life, with findings suggesting that circulating sex steroids are a crucial contributor to gender differences in serum Hcy [43–46]. However, along with previous studies, this study indicated that gender differences in serum Hcy exist, even when controlling for confounding variables.

Following gender separation, a significant positive correlation between patient age and Hcy was observed in both males and female PwP (*r*_s_ = 0.163, *p*=0.037; *r*_s_ = 0.366, *p* < 0.001, respectively). Serum Hcy levels are known to increase with age, which may be due to age-related impairment of associated enzymes and renal function [47]. In addition, the previous literature suggests that Hcy level is associated with levodopa usage, which routinely increases with disease duration. In the current cohort, LED (*r*_s_ = 0.278, *p*=0.001) and disease duration (*r*_s_ = 0.230, *p*=0.008) were significantly correlated with serum Hcy levels in male patients; however, this association was not observed in female patients. It also appeared that serum Hcy levels in male patients were predictive of worsening motor symptoms, with increases of 0.77 points on the MDS-UPDRS III for each additional 1 *µ*mol/L of serum Hcy. Previous studies have also suggested an association between increasing Hcy and occurrence of dyskinesias, a phenomenon which is thought to occur due to a disruption in striatal activity homeostasis [48]. Further, several studies have supported the notion that HHcy is predictive of deteriorating mobility and physical performances, indicated by poorer gait, and balance [49].

In addition to motor impairment, elevated serum Hcy levels have also been associated with cognitive impairment in Alzheimer's disease and vascular dementia [9, 19], as well as in PwP [28, 48]. Recent reports have suggested that elevated levels of Hcy stimulate oxidative injury in these cognitive disorders and may lead to neuroinflammation as a result of a burdened cerebrovascular system and damaged neuronal and vascular endothelial cells [9, 11, 15, 26, 38, 50]. While some research has reported no such association [24, 30], more recent studies have found that HHcy is implicated in aspects of cognitive dysfunction and have suggested a relationship with cognitive impairment in PwP [26,51–53]. Accordingly, the present study showed serum Hcy correlated with gender-specific impairment of cognition in female PwP. When controlling for confounding variables, it was found that serum Hcy was predictive of total ACE-R score, a significant finding only observed in female participants. These findings further support the notion that elevated Hcy levels do have a significant association with cognitive impairment but that this is gender-specific.

Findings from the present study emphasise that elevated Hcy levels correlate differentially with motor and cognitive measures in male and female PD patients, with increased Hcy being associated with worsening motor symptoms in males and poorer cognitive performance in females. Prior studies have found gender associations between the Hcy-related *MTHFR* polymorphism [23], environmental factors, and the impact that elevated Hcy has on various disease states and outcomes [39, 54, 55]. However, Christine et al.[2] recently reported that low levels of vitamin B12 were predictive of worsening motor outcome scores in PwP and that elevated Hcy was predictive of a greater cognitive decline. A large proportion of literature suggests that serum vitamin B12 concentrations are generally lower in men compared to women, thereby providing a potential mechanism whereby male patients are more susceptible to displaying motor impairment [56, 57]. In this study, both male patients and controls had a noticeably lower vitamin B12 level than women, although not to statistically significant levels (*p*=0.054). However, there was a significant difference in serum vitamin B12 in males with PD (Table 2, *p* < 0.001) but not in females with PD (Table 2, *p*=0.163), compared to healthy controls. This finding partially lends support to the notion that Christine et al.[2] reported low levels of vitamin B12 may be predictive of greater motor impairment and that elevated Hcy may be predictive of impaired cognitive function. Our study subsequently suggests that there may be a complex interplay between serum vitamin deficiencies and gender in determining the influence of elevated Hcy on the clinical phenotype of PD. However, it is also known that genetic variants may too influence phenotype variability within PD [23, 24, 58]. To our knowledge, there are no findings that report a mechanism by which female participants are more susceptible to cognitive impairments as a result of elevated Hcy, and none that explain why males with PD may be more prone to manifest movement difficulties rather than cognitive dysfunction.

## 5. Limitations

A number of limitations of the current study must be noted. To overcome possible selection bias, home-based PwPs were recruited sequentially from movement disorder clinics across Australia but did not include patients with more advanced PD who were no longer independent. As the study was cross-sectional in nature, we did not monitor changes in homocysteine levels over the course of the disease. As such, longitudinal studies are needed to explore the alterations of this serum biomarker during the disease progression in PD. Further, as the ACE-R does not include tests of executive function [36], our findings need to be confirmed using more comprehensive cognitive test protocols. Lastly, there are other known parameters that are related to Hcy levels, such as cerebrovascular disease burden, that were not able to be evaluated in this study. Therefore, future studies may consider examining these factors, which could potentially affect motor and cognitive scores in PD due to the impact that they have on serum Hcy levels.

## 6. Conclusions

The findings in this PD cohort indicate that elevated serum Hcy levels are differentially associated with motor and cognitive performance in male and female patients. After controlling for other variables, higher serum Hcy levels were predictive of more severe motor disability in males, whereas in females, higher levels were associated with poorer cognitive function and there was no association with motor status. We believe that this finding of a gender effect may help to explain previous discrepancies in the literature surrounding the utility of Hcy as a biomarker in PD. It is unclear whether HHcy plays a pathophysiological role in PD, or whether it is rather a surrogate marker of the underlying neurodegenerative process [50]. Nevertheless, the gender-specific differences found in the present study warrant further consideration in future larger studies in PD.

## Figures and Tables

**Table 1:** Baseline clinical characteristics of the PD cohort (*n*=205) used in this study.

Clinical characteristics	Mean (SD) or *n* (%)
Gender	
Male	128 (62.43%)
Female	77 (37.56%)
Age (years)	64.0 (9.38)
Disease duration (years)	8.9 (5.80)
LED (mg/day)	888.6 (587.9)
Deep brain stimulation	
Yes	37 (18.05%)
No	168 (91.95%)
MDS-UPDRS III score	19.56 (13.15)
H&Y score	1.69 (0.92; IQR = 1)
Total ACE-R score	85.72 (12.65)
Homocysteine (*µ*mol/L)	11.55 (4.06)
Vitamin B12 (pmol/L)	331.87 (184.54)
Serum folate (nmol/L)	31.59 (8.40)

**Table 2:** Serum markers divided according to gender.

Variable	Mean (SD)		
	Control	PD	Significance (*p* value; Cohen's *d*)
Homocysteine (*µ*mol/L)			
Male	10.36 (3.42)	12.23 (4.08)	*p* < *.001*; *d* = 0.49
Female	8.39 (2.39)	10.76 (5.58)	*p*=0*.009*; *d* = 0.55

Vitamin B12 (pmol/L)			
Male	311.01 (113.36)	294.71 (142.56)	*p* < *.001*; *d* = 0.13
Female	350.26 (110.70)	394.61 (223.40)	*p*=0.163; *d* = 0.25

Serum folate (nmol/L)			
Male	29.18 (8.12)	31.19 (8.66)	*p*=0.557; *d* = 0.24
Female	30.29 (7.36)	31.57 (8.27)	*p*=0.769; *d* = 0.16

**Table 3:** Spearman's (rho) correlation analysis between Hcy levels and disease-related variables in males and females with PD.

Variable	Male PwP (*n*=128)		Female PwP (*n*=77)	
	*r*_s_	*p* value	*r*_s_	*p* value
Age	0.163	**0.037**	0.366	**<0.001**
Disease duration	0.230	**0.008**	0.080	0.484
Total levodopa equivalent	0.278	**0.001**	0.211	0.062
MDS-UPDRS III	0.319	**<0.001**	0.197	0.083
Total ACE-R	−0.082	0.358	−0.449	**<0.001**

**Table 4:** Final multivariate model parameter estimates: predictors of UPDRS III score, when separated by gender.

Gender	Variable	*β* coefficient	Std. error	Significance
Female	Intercept	−19.934	11.910	0.094
	Disease duration	0.103	0.292	0.724
	Age at assessment	0.432	0.196	**0.027**
	Total levodopa equivalent	0.005	0.003	0.081
	Hcy	0.375	0.274	0.170

Male	Intercept	−15.341	7.106	0.031
	Disease duration	0.719	0.186	**<0.001**
	Age at assessment	0.354	0.105	**0.001**
	Total levodopa equivalent	−0.002	0.002	0.370
	Hcy	0.766	0.264	**0.004**

**Table 5:** Final multivariate model parameter estimates: predictors of ACE-R score, when separated by gender.

Gender	Variable	*β* coefficient	Std. error	Significance
Female	Intercept	95.513	8.946	0.000
	Disease duration	−0.437	0.218	**0.045**
	Age at assessment	0.029	0.148	0.847
	Total levodopa equivalent	−0.001	0.002	0.530
	Hcy	−0.473	0.204	**0.021**

Male	Intercept	105.810	7.930	0.000
	Disease duration	−0.366	0.208	0.078
	Age at assessment	−0.249	0.116	**0.033**
	Total levodopa equivalent	−0.002	0.002	0.312
	Hcy	0.016	0.293	0.955

## Data Availability

The data used to support the findings of this study are included within the article.

## Supplementary Materials

Supplementary Materials Supplementary Table 1. demographic variable comparisons between gender.
