# Supplementary material for: Elevated Serum Homocysteine Levels Have Differential Gender-Specific Associations with Motor and Cognitive States in Parkinson's Disease
Source: Parkinsons Dis. 2019 May 29;2019:3124295. doi: 10.1155/2019/3124295 (PMC6560330; doi:10.1155/2019/3124295)
Supplement: Supplementary Materials — Supplementary Table 1. demographic variable comparisons between gender. [file 3124295.f1.docx]

**Supplementary Table 1.** Demographic variable comparisons between gender

| Variable |  | Mean (SD) | Significance  (*p* value) |
| --- | --- | --- | --- |
| Age (years) | Male | 64.07 (10.47) | *p* =.769 |
|  | Female | 64.47 (7.65) |  |
| Disease duration (years) | Male | 9.36 (6.19) | *p* =.344 |
|  | Female | 8.50 (5.68) |  |
| LED (mg/day) | Male | 922.34 (616.51) | *p* =.260 |
|  | Female | 863.06 (619.31) |  |
| MDS-UPDRS III | Male | 21.96 (13.75) | ***p* =.001** |
|  | Female | 16.79 (13.88) |  |
| ACE-R | Male | 84.85 (13.47) | *p* =.193 |
|  | Female | 87.43 (10.35) |  |
